# Peri-Coronary Adipose Tissue Attenuation and Atrial Fibrillation Recurrence After Catheter Ablation: A Systematic Review and Meta-Analysis

**DOI:** 10.7759/cureus.114896

**Published:** 2026-08-20

**Authors:** Sandeep Guntuku, Priyank Chokshi, Siddharth Karipineni, Dishant Rakholia, Mounica Vorla, Narayana Varalakshmi Akula, Vimala Thambi, Pawel Lajczak, Muhammad F Ali, Sherif Eltawansy, Rajeev Vijay Kumar Menon

**Affiliations:** 1 Internal Medicine, Mamata Medical College, Khammam, IND; 2 Internal Medicine, Jersey City Medical Center, Jersey City, USA; 3 Internal Medicine, The Brooklyn Hospital Center, New York City, USA; 4 Internal Medicine, Nassau University Medical Center, East Meadow, USA; 5 Internal Medicine, Carle Foundation Hospital, Urbana, USA; 6 Internal Medicine, Harlem Hospital Center, New York, USA; 7 Internal Medicine, Medical University of Silesia, Katowice, POL; 8 Internal Medicine, Centerpoint Medical Center, Kansas City, USA; 9 Internal Medicine, Jersey Shore University Medical Center, Neptune City, USA; 10 Cardiology, AIG Hospitals, Hyderabad, IND

**Keywords:** atrial fibrillation, catheter ablation, coronary computed tomography angiography (ccta), coronary inflammation, meta-analysis, peri-coronary adipose tissue, post-ablation recurrence, pulmonary vein isolation, risk stratification

## Abstract

Background: Catheter ablation is an established rhythm-control strategy for atrial fibrillation (AF), but arrhythmia recurrence after the procedure remains a major clinical challenge. Reliable pre-procedural biomarkers reflecting the underlying inflammatory substrate may improve risk stratification and patient selection. Peri-coronary adipose tissue (PCAT) attenuation, measured on coronary computed tomography angiography (CCTA), is a noninvasive imaging marker of coronary perivascular inflammation. This study evaluated the association between PCAT attenuation and AF recurrence after catheter ablation.

Methods: We systematically searched PubMed, Embase, and the Cochrane Central Register of Controlled Trials through October 2024, following the Preferred Reporting Items for Systematic Reviews and Meta-Analyses (PRISMA) 2020 guidelines, for studies comparing PCAT attenuation between patients with and without AF recurrence after catheter ablation. Pooled mean differences (MDs) with 95% confidence intervals (CIs) were calculated separately for each coronary territory using random-effects models with restricted maximum likelihood estimation of the between-study variance and Knapp-Hartung-Sidik-Jonkman-corrected confidence intervals, an approach recommended when the number of available studies is small. DerSimonian-Laird estimates with Wald intervals are reported alongside for comparison. Standardized MDs were calculated in parallel. Heterogeneity was assessed using the I² statistic, and leave-one-out analysis was performed as a descriptive check.

Results: Three retrospective cohort studies enrolling 852 patients (239, 28%, with AF recurrence) met the inclusion criteria. PCAT attenuation was numerically higher in patients with AF recurrence across all three major coronary territories, but under the small-sample-corrected model, none of the associations reached statistical significance: right coronary artery (RCA)-PCAT (MD 2.62 HU; 95% CI −2.41 to 7.65), left anterior descending artery (LAD)-PCAT (MD 1.53 HU; 95% CI −1.45 to 4.50), and left circumflex artery (LCx)-PCAT (MD 2.48 HU; 95% CI −0.70 to 5.66). Corresponding standardized MDs were 0.30, 0.23, and 0.36. Conventional DerSimonian-Laird estimates with Wald intervals were nominally significant in all three territories despite identical point estimates, reflecting the degree to which precision is overstated when the between-study variance is estimated from three studies. Because each patient contributed correlated measurements across territories, no overall pooled estimate combining territories was calculated. Heterogeneity was moderate (I² 34-65%) but imprecisely estimated.

Conclusion: Pre-ablation PCAT attenuation was consistently higher in patients who subsequently experienced AF recurrence after catheter ablation, but the pooled differences of approximately 1.5-2.6 HU did not reach statistical significance once appropriate small-sample corrections were applied and are of a magnitude comparable to reported technical variability in PCAT measurement across CT acquisition protocols. The consistent direction of effect across three independent cohorts is of interest and supports further investigation, but these data do not establish PCAT attenuation as a predictor of post-ablation recurrence and provide no basis for clinical application. Prospective multicenter studies with standardized acquisition and segmentation protocols are needed.

## Introduction and background

Atrial fibrillation (AF) is the most common cardiac arrhythmia, resulting from abnormal electrical activity in the atria. This arrhythmia can be paroxysmal if it lasts less than seven days or persistent if it lasts more than seven days [1]. The risk of developing AF is approximately 25% after age 40 [2,3]. Its irregular rhythm disrupts normal blood flow, increasing turbulence and the risk of thrombus formation. If dislodged, the clot can embolize to the cerebral circulation, leading to a stroke [4]. There is also an increased risk of adverse cardiovascular outcomes, including heart failure, chronic kidney disease, and myocardial infarction (MI), which makes appropriate treatment and careful monitoring necessary [5-7].

Treatment modalities include rhythm control and rate control. Cardiac tissue ablation is considered the definitive treatment option for AF, but arrhythmia recurrence after the procedure remains common, particularly in patients with persistent AF [8,9]. Recurrence is more likely with increased left atrial volume or fibrosis, systemic diseases like hypertension or sleep apnea, concomitant heart conditions (e.g., mitral valve disease and hypertrophic cardiomyopathy), and longer AF duration, with persistent AF carrying a higher risk than paroxysmal AF [10]. Factors such as atrial remodeling, obesity, and inflammation have been shown to play a substantial role in AF recurrence [11-13].

Epicardial adipose tissue (EAT) is a fat layer surrounding the myocardium, which is known to have a role in atrial remodeling, conduction pathway remodeling, and inflammatory damage [14]. Peri-coronary adipose tissue (PCAT) is an extension of EAT. In this study, we focus on PCAT attenuation as a marker of post-ablation AF recurrence. There is evidence of an association between inflammation and AF progression [11,14], but the role of PCAT attenuation in AF recurrence following ablation remains unclear, as data are limited [15,16]. To our knowledge, this is the first systematic review and meta-analysis to assess the relationship between PCAT attenuation and AF recurrence after catheter ablation.

## Review

Methods

This systematic review and meta-analysis was conducted in accordance with the Cochrane Handbook for Systematic Reviews of Interventions and reported in accordance with the Preferred Reporting Items for Systematic Reviews and Meta-Analyses (PRISMA) 2020 guidelines [17,18].

Literature Search

Two independent reviewers searched PubMed, Embase, and the Cochrane Central Register of Controlled Trials from inception through October 2024 using MeSH terms and free-text keywords: "pericoronary adipose tissue", "peri-coronary adipose tissue attenuation", "PCAT", "epicardial fat", "atrial fibrillation", "catheter ablation", "pulmonary vein isolation", and "recurrence". The PICO framework is presented in Table 1. The full database-specific search strategies and the number of records retrieved from each source are presented in Table 2. Reference lists of included studies were manually screened. Discrepancies were resolved by consensus with a third reviewer.

**Table 1:** Population, Intervention, Comparison, Outcome (PICO) framework

Parameter	Definition
Population (P)	Adults ≥18 years with paroxysmal or persistent AF undergoing first-time catheter ablation with pre-procedural CCTA
Exposure (E)	Pre-ablation PCAT attenuation (HU) measured on CCTA in the RCA, LAD, and LCx territories
Comparison (C)	Patients without AF recurrence after catheter ablation
Outcome (O)	AF recurrence after catheter ablation defined as any atrial tachyarrhythmia lasting >30 seconds after a 3-month blanking period. Effect measures: mean difference in PCAT attenuation (HU) with 95% CI (primary); standardised mean difference (secondary)
Study design (T)	Observational cohort studies published up to October 2024

**Table 2:** Database search strategy All databases were searched from inception to October 2024. The records shown are the numbers retrieved from each source before deduplication. CENTRAL, Cochrane Central Register of Controlled Trials; MeSH, Medical Subject Headings; tiab, title/abstract field; exp, exploded subject heading

Database	Search strategy	Records
PubMed	("pericoronary adipose tissue"[tiab] OR "peri-coronary adipose tissue"[tiab] OR "PCAT"[tiab] OR "fat attenuation index"[tiab] OR "epicardial adipose tissue"[tiab] OR "epicardial fat"[tiab]) AND ("atrial fibrillation"[MeSH] OR "atrial fibrillation"[tiab]) AND ("catheter ablation"[MeSH] OR "radiofrequency ablation"[tiab] OR "cryoballoon"[tiab] OR "pulmonary vein isolation"[tiab]) AND ("recurrence"[MeSH] OR "recurrence"[tiab])	412
Embase	('pericoronary adipose tissue':ab,ti OR 'fat attenuation index':ab,ti OR 'pcat':ab,ti OR 'epicardial adipose tissue'/exp) AND ('atrial fibrillation'/exp) AND ('catheter ablation'/exp OR 'radiofrequency ablation':ab,ti OR 'cryoballoon':ab,ti OR 'pulmonary vein isolation':ab,ti) AND ('recurrence'/exp)	583
Cochrane CENTRAL	("pericoronary adipose tissue" OR "PCAT" OR "fat attenuation index" OR "epicardial adipose tissue") AND ("atrial fibrillation") AND ("catheter ablation" OR "radiofrequency ablation" OR "cryoballoon" OR "pulmonary vein isolation") AND ("recurrence")	26

Eligibility Criteria

Studies were eligible if they enrolled adults ≥18 years with paroxysmal or persistent AF undergoing catheter ablation, performed pre-ablation coronary computed tomography angiography (CCTA) reporting PCAT attenuation values (HU) for ≥1 coronary territory, and compared PCAT attenuation between patients with and without AF recurrence. Studies were excluded if they were case reports, editorials, or reviews; lacked a comparator group; or reported PCAT volume rather than attenuation. This review was not prospectively registered.

Outcome Definition and Exposure Measurement

Across the included studies, AF recurrence was defined consistently as any atrial tachyarrhythmia (AF, atrial flutter, or atrial tachycardia) lasting more than 30 seconds, documented on 12-lead ECG or 24-hour Holter monitoring after a three-month post-ablation blanking period. Monitoring schedules were broadly comparable, comprising scheduled outpatient visits with ECG and Holter assessment at three-month intervals during the first year, supplemented by event recording where symptoms occurred.

PCAT attenuation was measured on pre-ablation CCTA as the mean CT attenuation of perivascular fat (voxels within the −190 to −30 HU range) located within a radial distance from the outer vessel wall equal to the diameter of the target vessel. The proximal 40 mm segments of the left anterior descending and left circumflex arteries and the proximal 10-50 mm segment of the right coronary artery (RCA) (excluding the first 10 mm to avoid aortic-wall influence) were analyzed, in accordance with the standard published approach to perivascular fat attenuation measurement. Segmentation was performed using semi-automated dedicated software in each study.

The primary outcome was the difference in PCAT attenuation between patients with and without AF recurrence after catheter ablation, expressed as a mean difference with 95% confidence intervals.

Data Extraction and Statistical Analysis

Two reviewers independently extracted data on study design, sample size, AF type, ablation technique, PCAT attenuation values per territory, recurrence definition, and follow-up duration. Pooled mean differences (MDs) with 95% confidence intervals (CI) were estimated using inverse-variance random-effects models with restricted maximum likelihood estimation of the between-study variance. CIs were computed using the Knapp-Hartung-Sidik-Jonkman variance correction, which employs a t-distribution with k−1 degrees of freedom and is recommended when the number of included studies is small, as conventional Wald intervals assume the between-study variance is known and substantially understate uncertainty under these conditions. Estimates obtained using the DerSimonian-Laird method with Wald intervals are reported alongside for comparability with the wider literature. For Ma et al. [19], which reported per-territory values as medians with interquartile ranges, means and standard deviations were estimated using the method of Wan et al. [20]; the original medians and interquartile ranges are provided in Appendix A. Because each patient contributed correlated PCAT measurements for the RCA, left anterior descending artery (LAD), and left circumflex artery (LCx) territories, effect sizes were pooled within each territory separately, and no single estimate combining all territories was derived to avoid violating the independence assumption of standard meta-analysis. For the same reason, no formal test for differences between territories was performed. Standardized mean differences (Hedges' g) were calculated alongside mean differences to permit comparison against within-study measurement variability. Heterogeneity was quantified using I² and Cochran's Q, both of which are imprecisely estimated at this number of studies. Statistical significance was set at p < 0.05. Leave-one-out analyses are presented as a descriptive check rather than as a formal test of robustness, since with three studies each omission leaves a two-study synthesis. Prediction intervals were not estimated, as they are not interpretable with three studies, and formal assessment of small-study effects such as funnel-plot asymmetry testing was not meaningful and was therefore not performed. All analyses were conducted using R version 4.6.0 with the 'meta' package (version 8.5-0). The full analysis code is provided in Appendix B.

Risk-of-Bias Assessment

Methodological quality was assessed independently by two reviewers using the ROBINS-I tool [21], evaluating seven domains: confounding, participant selection, classification of interventions, deviations from intended interventions, missing data, measurement of outcomes, and selection of the reported result. In accordance with the ROBINS-I algorithm, the overall judgement for each study was taken as the least favourable rating across the seven domains. Disagreements were resolved by consensus.

Results

Study Selection

**Figure 1:**
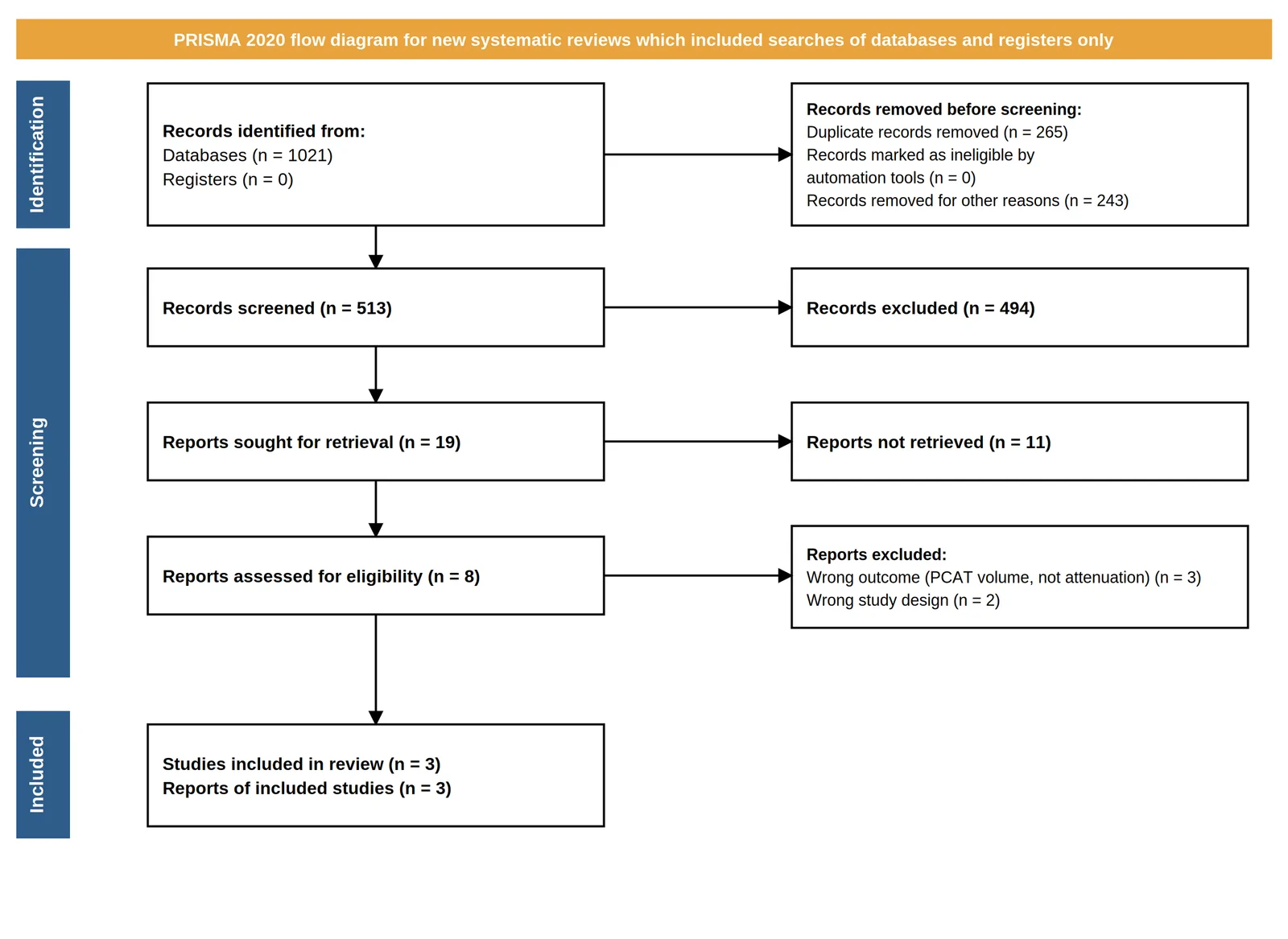
Preferred Reporting Items for Systematic Reviews and Meta-Analyses (PRISMA) flowchart

The systematic search identified 1,021 records. After removal of duplicates and ineligible records, title and abstract screening, and full-text review, three retrospective cohort studies met all eligibility criteria and were included [15,16,19]. The PRISMA flow diagram is shown in Figure 1.

Study and Patient Characteristics

The three included studies enrolled 852 patients undergoing catheter ablation for AF, of whom 239 (28.1%) experienced AF recurrence during follow-up. All were retrospective single-center cohort studies from Asia, two from China and one from Japan. Ablation techniques included radiofrequency catheter ablation in Wang et al. [15] and Ma et al. [19], and second-generation cryoballoon ablation in Nogami et al. [16]. Follow-up was fixed at 12 months in Wang et al. [15] and Ma et al. [19], whereas Nogami et al. [16] reported a median follow-up of 26 months (interquartile range 19-42 months). All studies reported PCAT attenuation for the RCA, LAD, and LCx territories. Eligibility criteria with respect to coronary artery disease differed between cohorts: Wang et al. [15] excluded patients with a history of coronary heart disease, whereas Nogami et al. [16] and Ma et al. [19] did not. Baseline characteristics were broadly comparable between the recurrence and non-recurrence groups in most respects, although several differences were reported: statin use and prior stroke were more frequent among patients with recurrence in Nogami et al. [16], and heart failure and left atrial diameter were greater in the recurrence group in Wang et al. [15]. Baseline patient characteristics for each study are presented in Table 3, and the characteristics of the included studies are presented in Table 4.

**Table 3:** Baseline characteristics of the included studies Values are presented as reported in the source publications: median [interquartile range], mean ± standard deviation, or n (%). PCAT attenuation values for Ma et al. [19] were reported as medians with interquartile ranges and were converted to means and standard deviations using the method of Wan et al. [20]; the originally reported values are provided in Appendix A. Nogami et al. [16] and Wang et al. [15] report current smoking, whereas Ma et al. [19] report smoking history. Male sex for Wang et al. [15] was derived from the reported female counts, and the proportion receiving RAAS blockers in the recurrence group was recalculated from the reported count and group denominator. Wang et al. [15] excluded patients with a history of coronary heart disease; Nogami et al. [16] did not exclude such patients but did not report prevalence. AAD, antiarrhythmic drug; BMI, body mass index; HU, Hounsfield units; LA, left atrium; LAD, left anterior descending artery; LCx, left circumflex artery; LVEF, left ventricular ejection fraction; N.A., not available; PCAT, peri-coronary adipose tissue; RAAS, renin-angiotensin-aldosterone system; RCA, right coronary artery; TIA, transient ischemic attack

Characteristic	Nogami et al. [16]		Wang et al. [15]		Ma et al. [19]	
	AF recurrence (n = 90)	No AF recurrence (n = 274)	AF recurrence (n = 102)	No AF recurrence (n = 197)	AF recurrence (n = 47)	No AF recurrence (n = 142)
Age, years	66 (55-71)	65 (58-70)	61.0 ± 11.3	60.2 ± 10.8	63 (57-71)	63 (56-66)
Male sex, n (%)	61 (67.8%)	190 (69.3%)	59 (57.8%)	117 (59.4%)	31 (66.0%)	74 (52.1%)
BMI, kg/m²	24.3 (22.4-26.3)	24.3 (22.1-26.3)	25.9 ± 3.6	25.3 ± 3.3	24.46 (23.44-26.43)	26.02 (23.59-28.23)
Hypertension, n (%)	53 (58.9%)	146 (53.7%)	60 (58.8%)	104 (52.8%)	28 (59.6%)	81 (57.0%)
Diabetes mellitus, n (%)	8 (8.9%)	36 (13.2%)	27 (26.5%)	55 (27.9%)	11 (23.4%)	20 (14.1%)
Prior stroke/TIA, n (%)	11 (12.2%)	13 (4.8%)	24 (23.5%)	31 (15.7%)	5 (10.6%)	25 (17.6%)
Heart failure, n (%)	3 (3.3%)	12 (4.4%)	23 (22.5%)	22 (11.2%)	N.A.	N.A.
Dyslipidaemia, n (%)	32 (35.6%)	127 (46.4%)	43 (42.2%)	70 (35.5%)	5 (10.6%)	31 (21.8%)
Smoking, n (%)	6 (6.7%)	34 (12.4%)	24 (23.5%)	38 (19.3%)	8 (17.0%)	23 (16.2%)
Coronary artery disease, n (%)	Not reported	Not reported	Excluded by design	Excluded by design	32 (68.1%)	75 (52.8%)
On statin therapy, n (%)	30 (33.3%)	57 (20.8%)	38 (37.3%)	60 (30.5%)	24 (51.1%)	85 (59.9%)
On RAAS blockers, n (%)	38 (42.2%)	96 (35.0%)	32 (31.4%)	47 (23.9%)	7 (14.9%)	24 (16.9%)
Pre-ablation AAD, n (%)	57 (63.3%)	154 (56.2%)	67 (65.7%)	113 (57.4%)	N.A.	N.A.
LA diameter, mm	40 (36-43)	38 (34-41)	41.0 ± 6.0	38.3 ± 6.0	35.91 ± 4.87	35.91 ± 4.83
LVEF, %	67 (63-70)	67 (63-71)	60.1 ± 6.5	60.6 ± 6.6	67.70 (63.70-70.99)	65.50 (61.20-70.10)
RCA-PCAT, HU	−68.62 ± 9.25	−71.47 ± 9.78	−74.0 ± 9.0	−78.4 ± 8.8	−86.33 ± 7.65	−86.67 ± 8.24
LAD-PCAT, HU	−68.82 ± 8.03	−71.08 ± 7.36	−74.8 ± 8.2	−77.0 ± 8.7	−81.83 ± 4.97	−82.00 ± 7.49
LCx-PCAT, HU	−63.84 ± 6.99	−65.71 ± 7.34	−72.1 ± 7.1	−73.6 ± 7.9	−77.17 ± 3.44	−81.00 ± 5.99

**Table 4:** Characteristics of the included studies CAD eligibility refers to whether patients with known coronary artery disease were excluded at enrollment. Wang et al. [15] excluded patients with a history of coronary heart disease; Nogami et al. [16] and Ma et al. [19] did not. AF, atrial fibrillation; CAD, coronary artery disease; IQR, interquartile range; LAD, left anterior descending artery; LCx, left circumflex artery; PCAT, peri-coronary adipose tissue; RCA, right coronary artery

Study	Study design	Country	N (total)	N (recur.)	N (no recur.)	AF type	CAD eligibility	Ablation technique	Follow-up	PCAT territories
Nogami et al. [16]	Retrospective cohort	Japan	364	90	274	Paroxysmal and persistent (323 paroxysmal, 41 persistent)	Not excluded	Second-generation cryoballoon ablation	26 months (median, IQR 19–42)	RCA, LAD, LCx
Wang et al. [15]	Retrospective cohort	China	299	102	197	Paroxysmal and persistent (172 paroxysmal, 127 persistent)	Excluded	Radiofrequency catheter ablation	12 months (fixed)	RCA, LAD, LCx
Ma et al. [19]	Retrospective cohort	China	189	47	142	Paroxysmal and persistent (151 paroxysmal, 38 persistent)	Not excluded	Radiofrequency catheter ablation	12 months (fixed)	RCA, LAD, LCx

Risk of Bias

Overall risk of bias was judged low in Wang et al. [15] and Nogami et al. [16] and moderate in Ma et al. [19], the latter reflecting a moderate rating in the domain of outcome measurement owing to potential subjective variability in PCAT segmentation. No study was judged to be at serious or critical risk of bias. The principal residual concerns across all three cohorts were confounding, as left atrial size, AF burden, and antiarrhythmic drug use were not uniformly reported, and selection bias inherent to retrospective single-center designs. Full domain-level judgements are shown in Appendix C.

PCAT Attenuation and AF Recurrence by Territory

**Figure 2:**
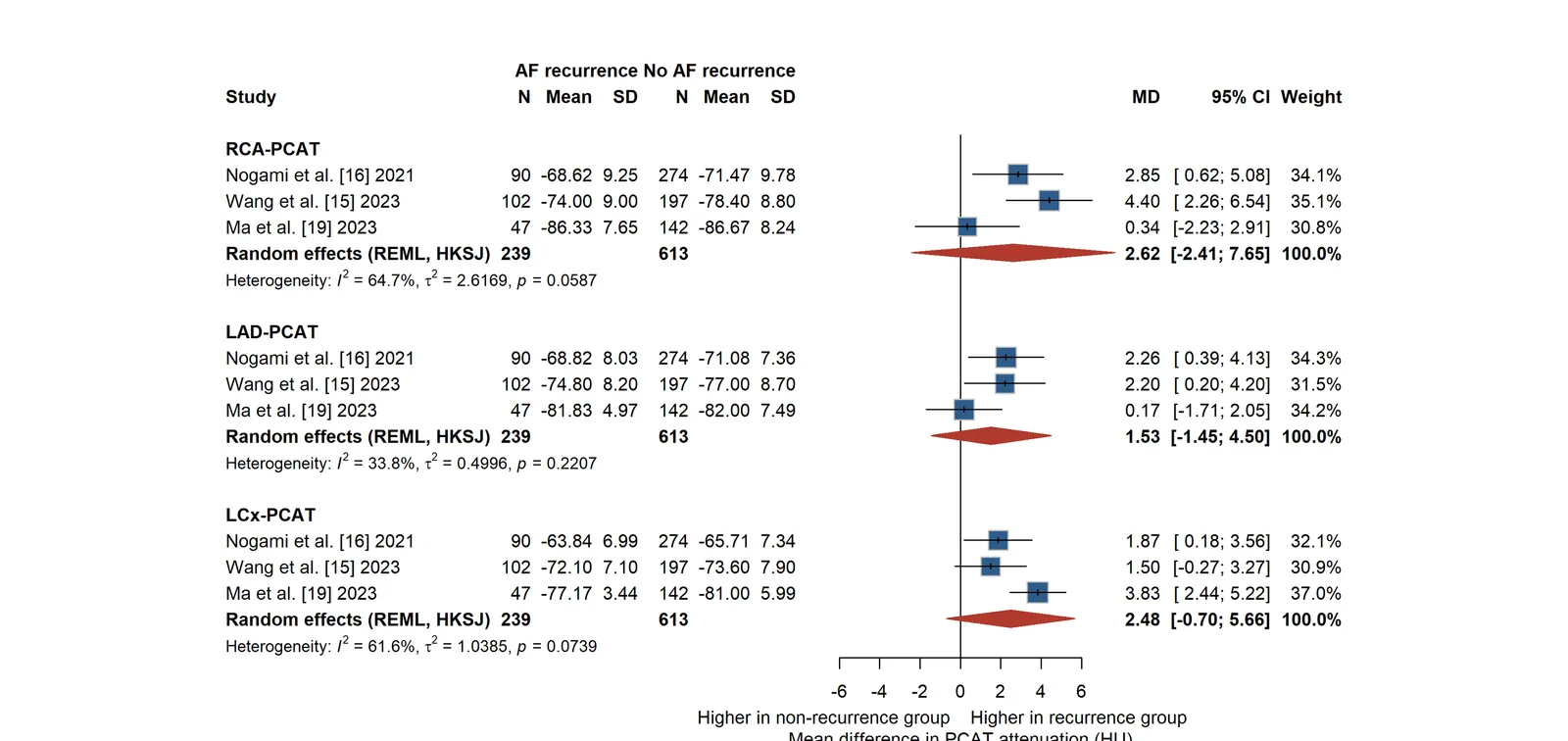
Forest plot of the mean difference in peri-coronary adipose tissue (PCAT) attenuation between patients with and without atrial fibrillation recurrence after catheter ablation, stratified by coronary territory. Studies are Nogami et al. [16], Wang et al. [15], and Ma et al. [19]. Blue squares represent study-level mean differences with 95% confidence intervals, sized by weight. Red diamonds represent the pooled random-effects estimate for each territory, calculated using restricted maximum likelihood with the Knapp-Hartung-Sidik-Jonkman variance correction. Positive values indicate higher (less negative) PCAT attenuation in patients with recurrence. Because each patient contributed correlated measurements across all three territories, results are pooled within territories only; no overall estimate and no formal comparison between territories was calculated. HU, Hounsfield units; RCA, right coronary artery; LAD, left anterior descending artery; LCx, left circumflex artery; MD, mean difference; CI, confidence interval

Because per-territory measurements within each patient are correlated, results are reported separately by territory rather than as a single combined estimate. Territory-specific findings are presented in Figure 2.

PCAT attenuation was numerically higher in the recurrence group in every territory in every included cohort, with all nine study-level mean differences positive in direction. Under the primary random-effects model with small-sample-corrected confidence intervals, none of the three pooled associations reached statistical significance: RCA-PCAT 2.62 HU (95% CI −2.41 to 7.65; p = 0.15), LAD-PCAT 1.53 HU (95% CI −1.45 to 4.50; p = 0.16), and LCx-PCAT 2.48 HU (95% CI −0.70 to 5.66; p = 0.08). Corresponding standardized mean differences were 0.30, 0.23, and 0.36, respectively, all within the range conventionally regarded as small.

Under the conventional DerSimonian-Laird approach with Wald intervals, all three territories were nominally significant: RCA-PCAT 2.62 HU (95% CI 0.38-4.87), LAD-PCAT 1.53 HU (95% CI 0.17-2.89), and LCx-PCAT 2.48 HU (95% CI 0.98-3.98). Point estimates were identical to two decimal places under both approaches, and the choice of between-study variance estimator had negligible influence on the result. The divergence between the two sets of intervals is therefore attributable entirely to the variance correction, and illustrates the degree to which precision is overstated when the between-study variance is estimated from three studies.

At the level of individual cohorts, Wang et al. [15] reported the largest RCA difference and identified RCA-PCAT as independently associated with recurrence, but found no significant difference in LCx-PCAT. Ma et al. [19] reported the largest LCx difference and identified LCx-PCAT as the strongest independent CT-radiomic predictor in that cohort (AUC 0.722; cut-off −81.5 HU), while reporting the smallest differences in the RCA and LAD territories. Nogami et al. [16] found a three-vessel PCAT attenuation index to be the strongest measure (cut-off −67.69 HU). It should be noted that Ma et al. [19] was the only cohort for which means and standard deviations were derived from reported medians and interquartile ranges [20], and that its comparatively narrow standard deviations gave it the largest weight in the LCx analysis.

Heterogeneity was moderate across territories, with I² values of 65%, 34%, and 62% for the RCA, LAD, and LCx, respectively, although these estimates are themselves imprecise at this number of studies.

Leave-one-out results are presented in Appendices D, E, and F for descriptive completeness. With three studies, each omission reduces the synthesis to two studies, and the resulting estimates are too unstable to support inference about the comparative reliability of any territory; they should not be interpreted as a formal robustness assessment.

Discussion

AF arises from complex pathophysiology in which obesity, inflammation, and coronary artery disease each contribute. Obesity is a major modifiable risk factor independent of obstructive sleep apnea or metabolic syndrome [22,23], and higher body mass index is associated with increased post-ablation recurrence, with one dose-response analysis reporting a 13% rise in recurrence risk per five-unit BMI increase [12]. Inflammation is increasingly recognized as central not only to AF incidence but also to recurrence after ablation [24,25]; elevated pre-procedural C-reactive protein and white-cell counts have been linked to unsuccessful cardioversion and to recurrence after ablative procedures [26,27]. These observations motivate interest in imaging markers that capture the inflammatory substrate directly, which is the rationale underlying the present analysis.

Adipose tissue secretes both endocrine proteins and pro-inflammatory mediators, producing local and systemic inflammatory effects that vary by anatomical site; visceral and mesenteric depots, for example, secrete substantially more interleukin-6 in obese individuals [28]. Because systemic inflammatory markers may not reflect the degree of local impact, attention has turned to depot-specific markers, such as epicardial and pericoronary adipose tissue [29,30]. EAT is a metabolically active fat layer between the myocardium and epicardium that secretes proinflammatory adipokines, contributing to inflammation, atrial fibrosis, and electrical remodeling that may promote the initiation and perpetuation of AF [14]. PCAT is an anatomical extension of EAT encroaching on the coronaries and pulmonary veins, and through paracrine and vasocrine signaling can influence the vascular regions it surrounds [31,32]. The PCAT attenuation index estimates coronary perivascular inflammation on CTA by detecting a shift in attenuation around inflamed coronaries from the lipid toward the aqueous phase, manifesting as less negative HU values [33,34]. Its role in coronary artery disease is established, with patients who have sustained MI exhibiting markedly thicker PCAT than healthy subjects and attenuation correlating with age, body mass index, glucose and triglyceride levels, and peak troponin I [35], whereas its significance in arrhythmias remains comparatively underexplored.

To our knowledge, this is the first meta-analysis to evaluate the association between PCAT attenuation and AF recurrence after ablation, pooling three retrospective cohorts comprising 852 patients. The direction of association was strikingly consistent: attenuation was higher in the recurrence group in every coronary territory in every included cohort, with all nine study-level estimates positive. The magnitude of the pooled differences, however, was modest at approximately 1.5 to 2.6 Hounsfield units, and once confidence intervals were computed using a method appropriate to the small number of available studies, none of the three territory-specific associations reached statistical significance. This is a meaningful distinction. Consistency of direction across independent cohorts is a genuine observation that warrants further study, but it is not the same as a demonstrated effect, and with three studies the data cannot distinguish a real association of this size from chance.

The modest magnitude of the differences also deserves comment in its own right. The pooled estimates correspond to standardized mean differences of 0.23 to 0.36, conventionally regarded as small, and the absolute differences of 1.5 to 2.6 HU are of a similar order to the variation in PCAT attenuation attributable to differences in tube voltage, acquisition protocol, region-of-interest definition, and segmentation software. Since the three included cohorts used different scanners and analysis platforms, a difference of this size cannot confidently be separated from technical variability, and this constrains what can be inferred even setting the question of statistical significance aside.

Apparent differences between territories should be interpreted with particular caution. Ma et al. [19] identified LCx-PCAT attenuation and the EAT volume index as independent correlates of recurrence in their cohort (area under the curve (AUC) 0.722 and 0.63 respectively, with an LCx cut-off of −81.5 HU and an EAT volume index above 81.07 mL/m²), suggesting that attenuation outperformed volume-based parameters. By contrast, Wang et al. [15] found no significant difference in LCx-PCAT attenuation but identified RCA-PCAT as independently associated with recurrence and as improving their clinical model. Nogami et al. [16] reported that a three-vessel PCAT attenuation index was an independent risk factor, with a cut-off of −67.69 HU, and that combining EAT volume with PCAT attenuation enhanced prediction. These divergent territory-level findings may reflect differences in cohort composition, ablation modality, follow-up duration, and CT acquisition and segmentation protocols. They may equally reflect sampling variation. Because all three territory-specific analyses draw on the same 852 patients, the three estimates are not independent, and no formal comparison between territories is statistically valid in this setting; we therefore make no claim that any one coronary territory carries a more informative signal than another.

Taken together, these data suggest a consistent direction of association between elevated PCAT attenuation and post-ablation AF recurrence, but do not establish that such an association exists independently of chance, nor that its magnitude exceeds measurement variability, nor that it would add to established clinical and imaging predictors. The attenuation thresholds reported across studies have not been externally validated and may not be generalizable, particularly as all included cohorts were drawn from Asian populations using differing CT acquisition and segmentation protocols. Adequately powered prospective, multicenter studies with standardized PCAT measurement are needed to determine whether PCAT attenuation provides independent, clinically actionable prognostic value.

Limitations

This synthesis is constrained primarily by the size of the available evidence base. Only three cohorts have reported per-territory PCAT attenuation stratified by post-ablation recurrence, and with so few studies the between-study variance is imprecisely estimated. We therefore adopted a conservative small-sample correction for the confidence intervals, under which the pooled estimates did not reach statistical significance. Given that three cohorts of this size provide limited power to detect differences of the magnitude anticipated for an imaging biomarker, this should be read as a statement about the maturity of the evidence rather than as evidence against an association, particularly since the direction of effect was positive in all nine study-level comparisons. For the same reason, leave-one-out results are presented descriptively, and prediction intervals and formal assessment of small-study effects were not estimated.

Clinical and technical heterogeneity between cohorts also warrants consideration. The studies differed in eligibility with respect to coronary artery disease, in ablation modality, and in follow-up duration, and all three were conducted in Asian populations, which may limit generalizability given reported population differences in adipose tissue distribution. PCAT attenuation is additionally sensitive to CT acquisition parameters, region-of-interest definition, and segmentation software, none of which were standardized across cohorts. One study reported values as medians and interquartile ranges, requiring conversion by a validated method that introduces some approximation. Our search was conducted through October 2024, and eligible cohorts published since are not represented here.

Finally, all included studies were observational, so residual confounding cannot be excluded, and no causal inference is possible. Whether PCAT attenuation adds prognostic value beyond established clinical and imaging predictors remains to be determined in adequately powered prospective multicenter studies with standardized measurement protocols.

## Conclusions

This systematic review and meta-analysis of three retrospective cohorts found that pre-ablation PCAT attenuation on CCTA was consistently higher in patients who subsequently experienced AF recurrence after catheter ablation, across all three major coronary territories, with pooled mean differences of approximately 1.5 to 2.6 HU. When appropriate small-sample corrections were applied, none of the territory-specific associations reached statistical significance, and the magnitude of the observed differences was comparable to the technical variability in PCAT measurement across the CT protocols used in the included studies. The consistency in the direction of effect across independent cohorts is of interest and warrants further investigation, particularly since PCAT attenuation can be derived from routine pre-procedural imaging without additional acquisition. However, the present evidence does not establish PCAT attenuation as a predictor of post-ablation recurrence and provides no basis for its clinical use. Prospective validation in adequately powered multicenter cohorts with standardized measurement protocols, together with assessment of incremental value over established clinical and imaging predictors, is required before any clinical integration.

## Figures and Tables

**Figure 3:**
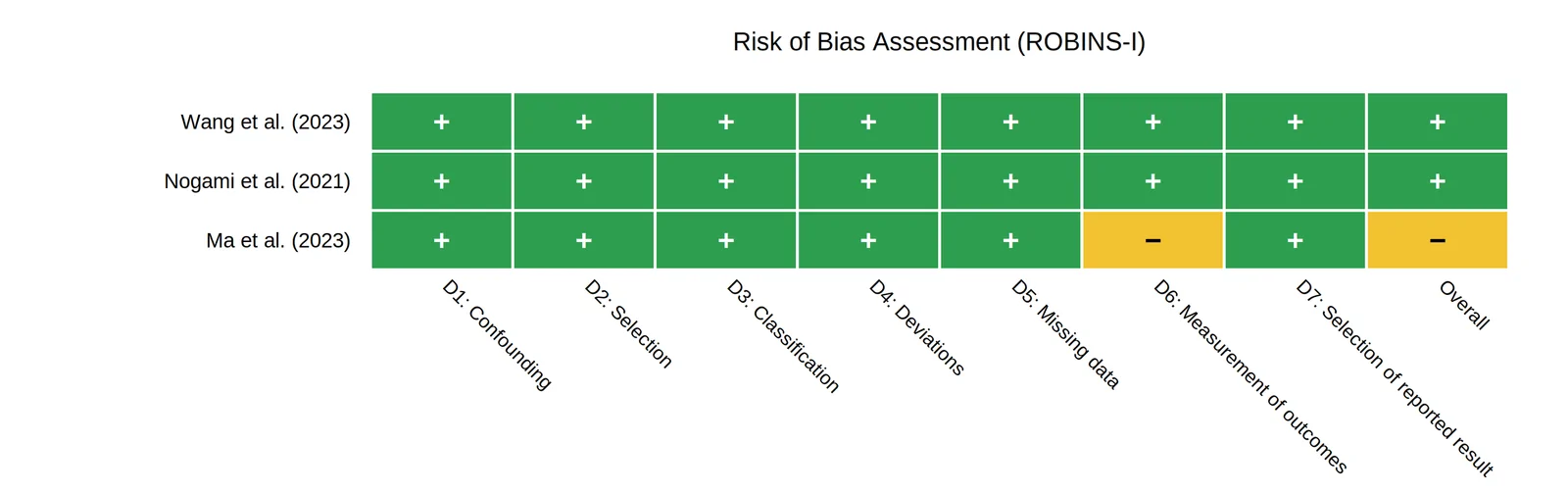
ROBINS-I risk-of-bias assessment for the included studies Wang et al. [15], Nogami et al. [16], and Ma et al. [19] were assessed using the ROBINS-I tool across seven domains [21]. Green (+) = low risk of bias; yellow (−) = moderate risk of bias. In accordance with the ROBINS-I algorithm, the overall judgement for each study is the least favourable rating across the seven domains. Ma et al. [19] received a moderate rating for domain 6 (measurement of outcomes) owing to potential subjective variability in PCAT segmentation and is therefore rated moderate overall; Wang et al. [15] and Nogami et al. [16] were rated low risk overall. No study was judged to be at serious or critical risk of bias. D1, confounding; D2, participant selection; D3, classification of interventions; D4, deviations from intended interventions; D5, missing data; D6, measurement of outcomes; D7, selection of the reported result; PCAT, peri-coronary adipose tissue; ROBINS-I, Risk Of Bias In Non-randomised Studies of Interventions

**Figure 4:**
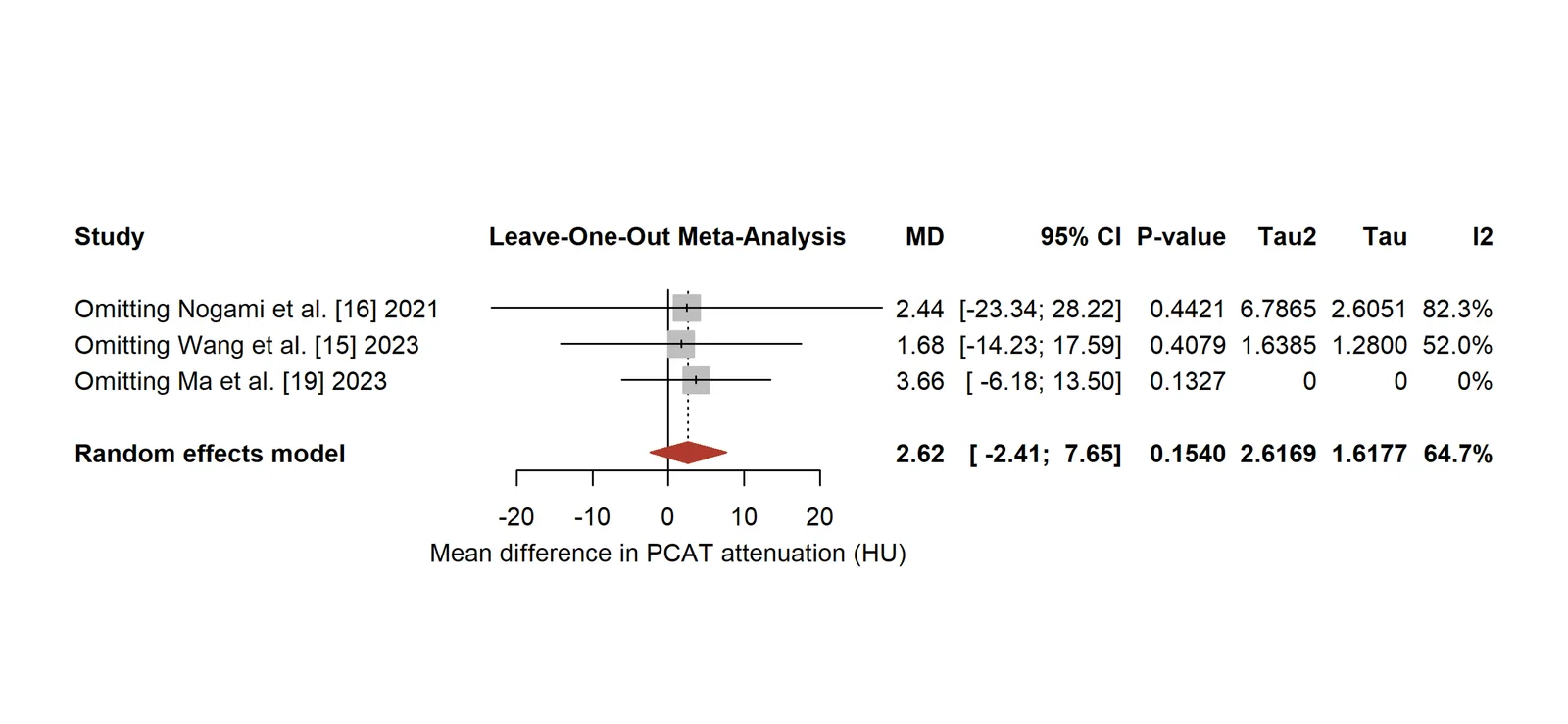
Leave-one-out analysis for RCA-PCAT attenuation Each row shows the pooled random-effects mean difference after sequential omission of one study, using the same model as the primary analysis. The bottom row is the full three-study estimate. With only three included studies, each omission leaves a two-study synthesis in which the between-study variance is estimated from a single degree of freedom, and the resulting confidence intervals are unstable in both directions, becoming implausibly wide when the two remaining estimates diverge and implausibly narrow when they nearly coincide. These results are presented for descriptive completeness only and do not constitute a formal robustness assessment. Includes Nogami et al. [16], Wang et al. [15], and Ma et al. [19]. CI, confidence interval; HU, Hounsfield units; MD, mean difference; PCAT, peri-coronary adipose tissue; RCA, right coronary artery

**Figure 5:**
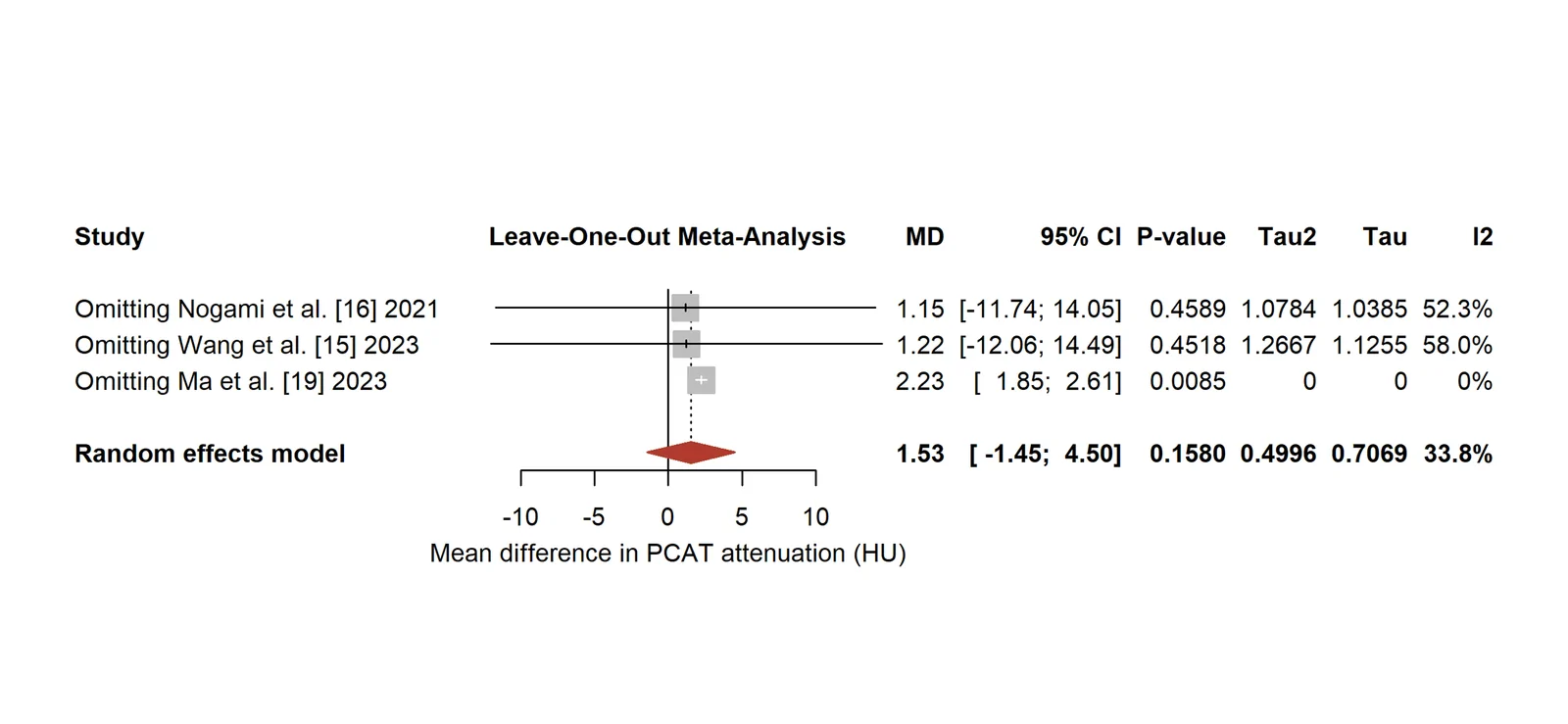
Leave-one-out analysis for LAD-PCAT attenuation Each row shows the pooled random-effects mean difference after sequential omission of one study, using the same model as the primary analysis. The bottom row is the full three-study estimate. With only three included studies, each omission leaves a two-study synthesis in which the between-study variance is estimated from a single degree of freedom, and the resulting confidence intervals are unstable in both directions. The interval obtained on omission of Ma et al. [19] is narrower than that of either contributing study and should not be interpreted as a precise estimate; it arises because the two remaining estimates nearly coincide, collapsing the observed between-study dispersion toward zero. These results are presented for descriptive completeness only and do not constitute a formal robustness assessment. Includes Nogami et al. [16], Wang et al. [15], and Ma et al. [19]. CI, confidence interval; HU, Hounsfield units; LAD, left anterior descending artery; MD, mean difference; PCAT, peri-coronary adipose tissue

**Figure 6:**
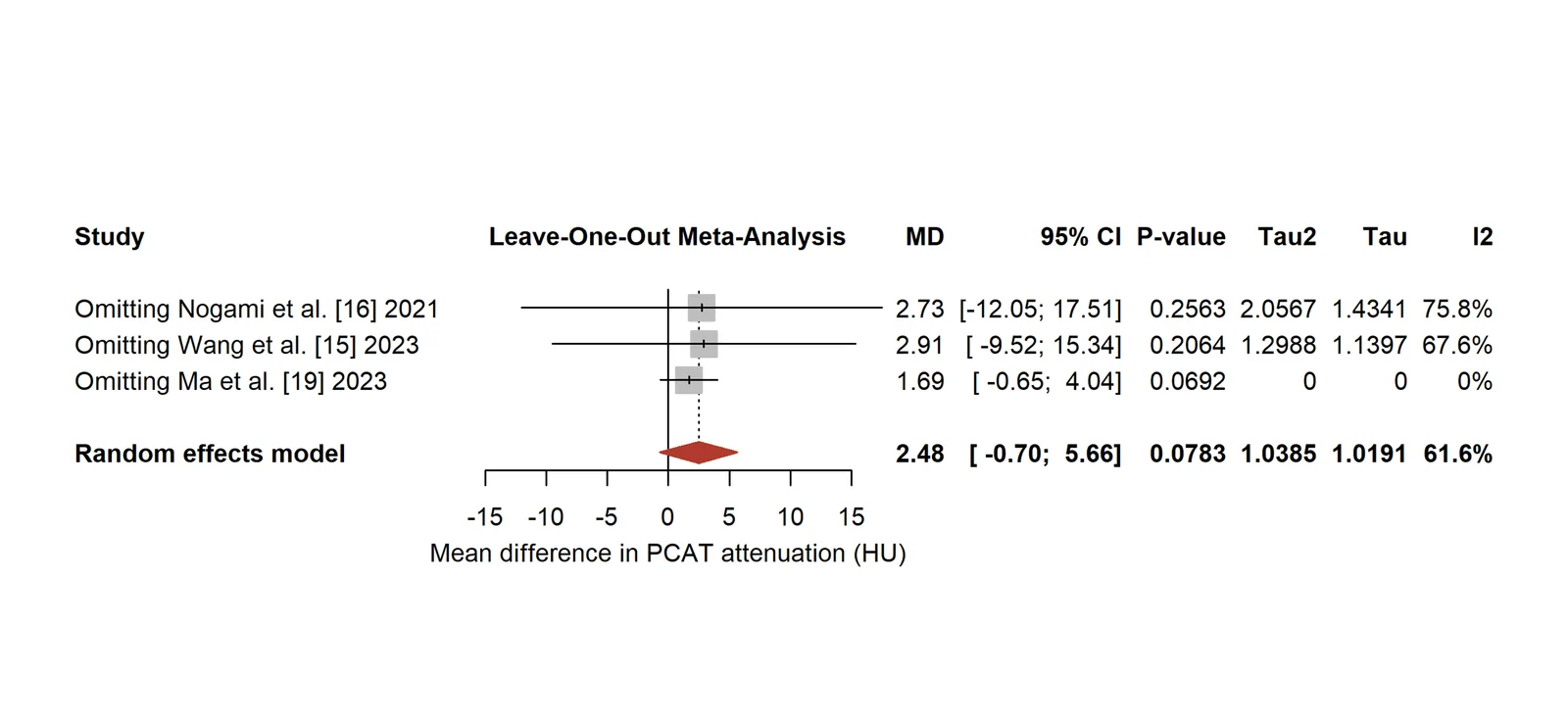
Leave-one-out analysis for LCx-PCAT attenuation Each row shows the pooled random-effects mean difference after sequential omission of one study, using the same model as the primary analysis. The bottom row is the full three-study estimate. With only three included studies, each omission leaves a two-study synthesis in which the between-study variance is estimated from a single degree of freedom, and the resulting confidence intervals are unstable in both directions, becoming implausibly wide when the two remaining estimates diverge and implausibly narrow when they nearly coincide. These results are presented for descriptive completeness only and do not constitute a formal robustness assessment. Includes Nogami et al. [16], Wang et al. [15], and Ma et al. [19]. CI, confidence interval; HU, Hounsfield units; LCx, left circumflex artery; MD, mean difference; PCAT, peri-coronary adipose tissue

## Appendices

Appendix A 

**Table 5:** Peri-coronary adipose tissue attenuation values as originally reported by Ma et al. [19) Values are median (interquartile range) as published. These were converted to means and standard deviations using the method of Wan et al. [20] for inclusion in the meta-analysis; the converted values are presented in Table 3. HU, Hounsfield units; LAD, left anterior descending artery; LCx, left circumflex artery; RCA, right coronary artery

Territory	AF recurrence (n = 47)	No AF recurrence (n = 142)
RCA-PCAT, HU	−86.00 (−91.50 to −81.50)	−87.00 (−92.00 to −81.00)
LAD-PCAT, HU	−82.00 (−85.00 to −78.50)	−82.00 (−87.00 to −77.00)
LCx-PCAT, HU	−77.00 (−79.50 to −75.00)	−81.00 (−85.00 to −77.00)

Appendix B 

Analysis Code

The following R code reproduces all pooled estimates, heterogeneity statistics, and leave-one-out analyses reported in this article. Analyses were performed in R version 4.6.0 with the meta package version 8.5-0. Values for Ma et al. [19] were derived from the published medians and interquartile ranges using the method of Wan et al. [20].

library(meta)

dat <- data.frame( terr = factor(rep(c("RCA","LAD","LCx"), each = 3), levels = c("RCA","LAD","LCx")), study = rep(c("Nogami 2021","Wang 2023","Ma 2023"), 3), n.e = rep(c(90,102,47), 3), mean.e = c(-68.62,-74.00,-86.33,-68.82,-74.80,-81.83,-63.84,-72.10,-77.17), sd.e = c(9.25,9.00,7.65,8.03,8.20,4.97,6.99,7.10,3.44), n.c = rep(c(274,197,142), 3), mean.c = c(-71.47,-78.40,-86.67,-71.08,-77.00,-82.00,-65.71,-73.60,-81.00), sd.c = c(9.78,8.80,8.24,7.36,8.70,7.49,7.34,7.90,5.99))

m.hk <- metacont(n.e, mean.e, sd.e, n.c, mean.c, sd.c, studlab = study, data = dat, sm = "MD", method.tau = "REML", method.random.ci = "HK", adhoc.hakn.ci = "", subgroup = terr, tau.common = FALSE, common = FALSE, random = TRUE, overall = FALSE, overall.hetstat = FALSE, test.subgroup = FALSE)

summary(m.hk)

m.dl <- update(m.hk, method.tau = "DL", method.random.ci = "classic") summary(m.dl)

m.smd <- update(m.hk, sm = "SMD") summary(m.smd)

forest(m.hk, overall = FALSE, overall.hetstat = FALSE, test.subgroup = FALSE, leftcols = c("studlab","n.e","mean.e","sd.e","n.c","mean.c","sd.c"), rightcols = c("effect","ci","w.random"), xlab = "Mean difference in PCAT attenuation (HU)")

for (t in c("RCA","LAD","LCx")) { d <- subset(dat, terr == t) m <- metacont(n.e, mean.e, sd.e, n.c, mean.c, sd.c, studlab = study, data = d, sm = "MD", method.tau = "REML", method.random.ci = "HK", adhoc.hakn.ci = "", common = FALSE, random = TRUE) print(metainf(m, pooled = "random")) }

sessionInfo()

Appendix C 

Appendix D 

Appendix E 

Appendix F
